# User Experiences in a Digital Intervention to Support Total Skin Self-examination by Melanoma Survivors: Nested Qualitative Evaluation Embedded in a Randomized Controlled Trial

**DOI:** 10.2196/39544

**Published:** 2023-02-13

**Authors:** Felicity Reilly, Nuha Wani, Susan Hall, Heather May Morgan, Julia Allan, Lynda Constable, Maria Ntessalen, Peter Murchie

**Affiliations:** 1 Institute of Applied Health Sciences University of Aberdeen Aberdeen United Kingdom; 2 Aberdeen Royal Infirmary National Health System Grampian Foresterhill Aberdeen United Kingdom; 3 Health Psychology Group University of Aberdeen Foresterhill Aberdeen United Kingdom; 4 Health Services Research Unit University of Aberdeen Foresterhill Aberdeen United Kingdom

**Keywords:** mobile apps, melanoma, early detection of cancer, qualitative interviews, cancer survivorship

## Abstract

**Background:**

Melanoma is a relatively common cancer type with a high survival rate, but survivors risk recurrences or second primaries. Consequently, patients receive regular hospital follow-up, but this can be burdensome to attend and not optimally timed to detect arising problems. Total skin self-examination (TSSE) supports improved clinical outcomes from melanoma via earlier detection of recurrences and second primaries, and digital technology has the potential to support TSSE. Recent research with app-based interventions aimed at improving the well-being of older adults has found that they can use the technology and benefit from it, supporting the use of digital health care in diverse demographic groups. Thus, the Achieving Self-directed Integrated Cancer Aftercare (ASICA) digital health care intervention was developed. The intervention provided melanoma survivors with a monthly prompt to perform a TSSE as well as access to a dermatology nurse who provided them with feedback on photographs and descriptions of their skin.

**Objective:**

We aimed to explore participants’ attitudes, beliefs, and experiences regarding TSSE practices. Furthermore, we explored how participants experienced technology and how it influenced their practice of TSSE. Finally, we explored the practical and technical experiences of ASICA users.

**Methods:**

This was a nested qualitative evaluation within a dual-center randomized controlled trial of the ASICA intervention. We conducted semistructured telephone interviews with the participants during a randomized controlled trial. The participants were purposively sampled to achieve a representative sample with representative proportions by age, sex, and residential geography. Interviews were transcribed verbatim and analyzed using a framework analysis approach applied within NVivo 12.

**Results:**

A total of 22 interviews were conducted with participants from both groups. In total, 40% (9/22) of the interviewed participants were from rural areas, and 60% (13/22) were from urban areas; 60% (13/22) were from the intervention group, and 40% (9/22) were from the control group. Themes evolved around skin-checking behavior, other people’s input into skin checking, contribution of health care professionals outside ASICA and its value, ideas around technology, practical experiences, and potential improvements. ASICA appeared to change participants’ perceptions of skin checking. Users were more likely to report routinely performing TSSE thoroughly. There was some variation in beliefs about skin checking and using technology for health care. Overall, ASICA was experienced positively by participants. Several practical suggestions were made for the improvement of ASICA.

**Conclusions:**

The ASICA intervention appeared to have positively influenced the attitudes and TSSE practices of melanoma survivors. This study provides important qualitative information about how a digital health care intervention is an effective means of prompting, recording, and responding to structured TSSE by melanoma survivors. Technical improvements are required, but the app offers promise for technologically enhanced melanoma follow-up in future.

**Trial Registration:**

ClinicalTrials.gov NCT03328247; https://clinicaltrials.gov/ct2/show/NCT03328247?term=ASICA&rank=1

**International Registered Report Identifier (IRRID):**

RR2-10.1186/s13063-019-3453-x

## Introduction

Melanoma is a cancer of the melanocyte or pigment cell of the skin. It is the fifth most common cancer in the United Kingdom, with >16,000 diagnoses conducted annually [1,2]. Melanoma accounts for 1% of all cancer-related deaths in the United Kingdom, equaling 2300 deaths annually [2]. Although melanoma can be fatal, the relative survival rate at 5 years is high, almost 90% in Scotland, with equivalent rates across the United Kingdom [3]. Once treated, there is a high chance of recurrence as well as the development of new primary melanoma [4]. Therefore, follow-up of patients with melanoma is very important. The high chance of recurrence, combined with high survival rates, leads to a large cohort of patients who require continued surveillance. This has led to patient care after melanoma becoming burdensome to both health services and patients [5].

Melanoma follow-up is largely based on regular hospital appointments where patients are physically examined. In addition, most guidelines recommend that patients perform the total skin self-examination (TSSE) in the intervals between hospital appointments [6-9]. Studies have shown that 62% of melanomas are first identified by patients themselves, with early detection being the key to improving clinical outcomes [9,10]. Regular TSSE by patients is thought to lead to earlier detection of melanoma [10,11]. However, the rate of skin checking after melanoma is similar to that of the general population as a whole, despite it being widely recommended [12].

Technology has become commonplace in daily life and is recognized as a potential solution to health care challenges, especially in rural areas [13-15]. A recent narrative review, including 15 studies of digital rural health care interventions, reported positive outcomes for patients and health care professionals, supporting their feasibility and potential [16]. In the United Kingdom, 55% of the population in the ≥65 age group owns a smartphone, and this number increases to 96% in younger groups [17], indicating that a high percentage of the population is familiar with this type of technology. Indeed, recent research with app-based interventions aiming to improve the well-being of older adults has found that both can use the technology and benefit from it [18,19]. Qualitative interviews undertaken with melanoma survivors suggest that, with appropriate training, smartphone apps offer an acceptable means to promote TSSE between routine follow-up appointments [20]. In a recent review in the United States, the authors reported the presence of 632 downloadable apps related to dermatology [21]. Of these, 94 focused on aiding people in monitoring, diagnosing, and treating skin conditions, such as skin cancer, providing evidence that a digital approach to management is not uncommon [21]. However, although smartphone apps can be helpful in cancer management, there is still a requirement for validation of their safety and utility [22,23].

The Achieving Self-directed Integrated Cancer Aftercare (ASICA) intervention aims to prompt, support, record, and respond to TSSE conducted by survivors of melanoma, and the development has been fully described elsewhere [20,24]. The intervention was delivered via a tablet and included access to personalized skin maps, as well as feedback from a dermatology nurse practitioner (DNP) on worrisome skin lesions. Using ASICA led to improvements in the participants’ well-being and TSSE adherence [25]. ASICA use also led to earlier detection and treatment of some relevant skin problems [26]. A detailed analysis of adherence suggested 3 patterns of adherence (close adherence, partial adherence, and nonadherence), as described in previous digital health adherence studies [27-29].

Qualitative studies can complement the quantitative data produced by randomized controlled trials (RCTs) by granting detailed insights into how participants have experienced the trial and intervention [30]. For the ASICA trial, a nested qualitative evaluation was conducted to facilitate understanding of how participants experienced the ASICA intervention and to attempt to explain the change in mechanisms underpinning the observed trial outcomes. In this nested study, we aimed to gather information on how the intervention and its underpinning technology could be improved ahead of the next stage of development and implementation, should ASICA be shown to have a positive impact on patient outcomes. In this nested qualitative evaluation, interviews were conducted with a representative sample of the participants in the ASICA trial. The interviews also formed part of the user-centered design approach, where users participated in every step of a product’s development to fully meet the needs of the users. The first aim of the study was to explore attitudes, beliefs, and TSSE practices in people treated for melanoma who had participated in the ASICA trial. The second aim of the study was to explore how users of ASICA experienced the technology and how it influenced their practice of TSSE. Third, we wanted to determine how ASICA users believed the intervention could be improved [24].

## Methods

### Study Design

This was a nested qualitative evaluation within a multicenter RCT of the ASICA intervention. Briefly, the ASICA intervention was an iteratively developed evidence-based app intervention to support and improve TSSE adherence and practice by people previously treated for melanoma using tablet-based technology. The app, hosted on Android tablets, uses animated instructional videos and monthly prompts to support TSSE. The app’s features included an individual digital skin map and a facility to send electronic reports of any skin concerns, including photographs, to a remote DNP. The DNP was a specialist nurse with expertise in clinical dermatology who reviewed the submitted information and assessed the patient.

Semistructured telephone interviews were guided by a predefined topic schedule (Multimedia Appendix 1). First, they explored the prior experiences of TSSE by trial participants, as well as their orientation toward technology, including its role in their health care and TSSE specifically. Furthermore, those who had experienced the ASICA intervention were asked about their practical experiences of the intervention and their views on how it might be improved. The app evaluation questions in the interview schedule were informed by a validated evaluation tool for health care apps [31]. This questionnaire was not presented to interview participants for completion during the trial; instead, its component questions were used as an aide-mémoire for the interviewer during the interviews.

### Sampling Strategy

The aim was to recruit approximately 10% of trial participants (intervention and control), up to a maximum of 30 participants across both sites. Purposive sampling was used when inviting participants to ensure the acquisition of multiple viewpoints representing the demographic range of the participants in the trial. The sampling sought representative proportions by age, sex, and residential geography. Age was chosen because it is a parameter that could influence familiarity with technology and its use to monitor one’s health [32]. Sex was used because it may influence adherence to health-checking behaviors [32]. Geography was chosen because the travel burden to attend follow-up appointments is well recognized for rural cancer survivors and, in theory, could influence an individual’s willingness to engage with telemedicine consultations [15]. The pool for potential participants in the nested qualitative evaluation was determined from those individuals who consented to be contacted for a subsequent interview at the point of recruitment. Demographic data supported a sampling framework to ensure that invitees represented the full range of age, sex, and rurality of trial participants. We also ensured that we recruited both intervention and control group participants for interviews. Because participants in the control group had not experienced the ASICA intervention, these interviews focused on their personal experiences of and orientation toward monitoring their skin during the study year.

### Recruitment

Eligible participants for the interview had completed 12 months in the ASICA trial, had not withdrawn from the trial, and had consented to be contacted for further research. Recruitment was carried out in 2 tranches, with a 6-month interval between the initiation of the ASICA trial in Aberdeen (March 2018) and Cambridge (November 2018). Potential interviewees were sent an invitation letter, a consent form, and a patient information leaflet describing the qualitative study. Participants who returned signed and correctly completed consent forms were contacted via telephone or email to arrange a suitable time for a call from one of the project interviewers for a telephone interview. All participants were given the opportunity to ask questions and seek further explanations of the qualitative study before proceeding. Recorded verbal consent was obtained before the start of each interview.

### Data Collection

The first tranche of telephone interviews with the Aberdeen participants (intervention and control) was conducted in April 2019. The second tranche of interviews with the Cambridge participants was conducted in November 2019. The sequential geographical interviewing pattern reflects the fact that recruitment was completed in Aberdeen sometime before that in Cambridge. The interviews were digitally recorded, anonymized, and transcribed verbatim by a professional transcribing service.

### Data Analysis

A preliminary thematic analysis was conducted on a sample of the Aberdeen interviews conducted in May 2019. Subsequently, all completed interviews were analyzed using a framework approach between April and July 2020 [33]. Professional transcriptions were uploaded to NVivo 12 (QSR International) for analysis [34]. Framework analysis was adopted because of its structured approach and for its utility in applied health research with well-defined questions and structured data, and in enabling comparison between several cases at once [35]. The first analytical step involves immersion. All transcripts were read and reread in sequence to enable familiarization with the data. This was particularly important because the analyst had not conducted or transcribed the interviews. A third reading identified the main themes and subthemes that were applied to the data and used to develop the initial analytical framework, which was checked, adapted, and agreed upon by the authors. Because the main analysis was completed by an analyst who had not conducted the interviews, the decision that data saturation had been reached was not straightforward. The pragmatic view was that, because similar themes were repeated in the Aberdeen and Cambridge interviews, the sample size was sufficient to capture the most important issues from the perspective of participants. A second analyst independently read and coded 3 interviews (approximately 10% of the sample) using the framework. Subsequent discussions enabled further minor adjustments and refinement of the framework. The final framework is available in Multimedia Appendix 2.

Subsequently, each interview transcript was coded in NVivo 12. All relevant data were sorted into identified themes and subthemes. The data were charted and interpreted thematically. Data from different respondents were compared, and an overview summarizing the data from each theme was created. Special consideration was given to the influence of individual demographics on their experiences, feelings, opinions, and suggestions for improvement. Finally, a full thematically structured narrative account supported by tabulated illustrative quotes was produced.

### Ethics Approval

For the ASICA study, ethics approval was given by the National Research Ethics Service Grampian Ethics committee on April 28, 2017 (reference number 17/NS/0040), and all participants gave written informed consent. This qualitative substudy received further ethics approval from the North of the Scotland Research Ethics Committee in February 2019 (reference number 17/NS/0040). This study was approved by the National Health System Grampian Research and Development in March 2019. All methods were carried out in accordance with Good Clinical Practices and the research governance and quality assurance policies and procedures of the University of Aberdeen.

## Results

### Sample Demographics

Of the 240 trial participants, 212 (88.3%) had consented to be contacted for further study. Invitations were sent to 32 participants (20 Aberdeen and 14 Cambridge), of whom 22 (13 Aberdeen and 9 Cambridge) replied that they were willing to participate in a telephone interview. Therefore, 22 telephone interviews, ranging in duration from 10 to 45 minutes, were completed and transcribed. In total, 60% (13/22) of the interviewed participants were in the intervention group, and 40% (9/22) were in the control group. The mean age of the participants was 56.3 (SD 14.7) years. In total, 45% (10/22) of the interviewees were female, and 55% (12/22) were male; 40% (9/22) of the interviewed participants were from rural areas, and 60% (13/22) were from urban areas. In total, 5% (1/22) of the interviewees lived in the second indices of multiple deprivation quintile, 10% (2/22) in the third quintile, 32% (7/22) in the fourth quintile, and 53% (12/22) in the fifth quintile. During coding, 6 main themes emerged: skin-checking behavior; friends and family; other health professionals; ideas around technology; “nuts and bolts”—practical experience of the ASICA trial; and finally, ASICA: the app impact, design, and usability. See Multimedia Appendix 2 for the full framework, themes, and subthemes.

### Theme 1: Skin-Checking Behavior

#### How Often and How Thoroughly Participants in Both Intervention and Control Groups Checked Their Skin During the ASICA Trial

More than three-quarters of the participants interviewed stated that they regularly examined their skin. Of those who did not, only 1 was in the ASICA intervention group. It appeared that living alone was a potentially determining factor, as well as not having someone to encourage, remind, and help them check awkward or difficult-to-see areas of the body. Individuals living alone tended to await for regular hospital follow-up appointments, feeling that they were sufficient for monitoring their condition. Those who regularly checked their skin mentioned a variety of approaches. The most common method was an unstructured look over. This idea ran through most interviews with participants, stating that it was part of the daily routine to “keep an eye” (Female, 49 years, control) on things:

I have a quick check to see there’s no other strange things happening and there isn’t.

Male, 76 years, control

I mean I do keep an eye on it, I don’t know if you would class it as examining.

Female, 48 years, control

Almost 90% of the intervention group stated that they used ASICA to aid skin checking, and most reported following the instructions provided, which were designed to facilitate high-quality skin examination. Two participants in the intervention group stated that they no longer used the app to check their skin; however, 1 described using what it had taught them initially to continue checking their skin using their own tablet:

Well I use the app, just look at the pictures and then look at the, look at the bits on my, bits on my skin, I usually get my husband to look at my back, backs of my legs and down my back and that.

Female, 47 years, intervention

The ipad is quite good at taking pictures close up as well, so that’s what we used, I find it a lot easier [than the study tablet], and it moves a lot easier to touch.

Male, 54 years, intervention

It was generally apparent from the interviews that skin checking was widely occurring and valued, but that the thoroughness and effectiveness of checking varied between individuals. All but 1 ASICA user reported making a conscious effort to check their skin regularly in a structured way, and approximately one-third stated that they had communicated concern about the study DNP using the app. Together, this suggests that ASICA can appropriately support skin checking, although further research is required to confirm this. Notably, 2 participants in the control group reported having used technology to digitally enhance their own skin checking and were tracking areas of concern with photos stored on their mobile phones.

#### Timing of Participants From Both Intervention and Control Groups Checking Their Skin

Although there were variations in how often and how thoroughly people checked their skin, the contexts in which the checks were conducted were consistent. Almost all mentioned checking their skin when they showered, with washing being a near-universal prompt:

If I’ve had a shower or whatever, I’ll sort of look, does that look different, or does it not.

Female, 47 years, intervention

The app users stated that the monthly reminders were useful prompts to remember to perform a structured skin check:

Every month I get a reminder to, to go over it on the app and check from top to toe.

Male, 61 years, intervention

Not only did the participants in the intervention group mention that the trial was triggering them to check skin but also a participant in the control group stated that taking part in the trial had influenced her to change her perceptions and personal practice with respect to skin checking.

The questionnaire probably acted as a reminder for me to do a little bit more.

Female, 49 years, control

#### Views About Knowledge and Skills Required to Perform a Skin Check From Both Intervention and Control Groups

Although all participants in the intervention group had received training on how to perform a TSSE as part of the intervention, reports of having received training to conduct TSSE in the past as part of usual care varied between individuals and study sites. Overall, 4 of the 6 Cambridge participants reported having received TSSE training, whereas only 3 of the 14 Grampian participants had received TSSE training before joining the study. Some participants had skin checking explained and demonstrated to them by a specialist, such as a nurse or a physician:

Fairly good instructions, and usually from the nurses more than the doctors, but on the skin checks I’ve had through the NHS, they, they’ve been quite good in terms of tutorials, so I’m quite happy in terms of what I’m looking at.

Female, 39 years, control

Less than one-quarter of the participants reported having received a leaflet explaining TSSE, whereas several participants reported no knowledge or education about TSSE.

I’ll just have a look, but no, never been told, whatever the, never been verbally told, there was a leaflet sometime back.

Male, 51 years, control

I haven’t been taught by anybody. I’ve been doing it for so long, I just know.

Male, 49 years, control

#### Beliefs About Skin Checking in Both Intervention and Control Groups

The value people placed on skin checking varied among individuals, but some dismissed its value, and it appeared to be rarely considered an active health improvement practice:

How do you learn to check your skin, you just look!

Female, 76 years, control

The relationship between previous melanoma and current health did not appear to be acutely perceived; only 1 participant mentioned previous melanoma when asked about their general health. Some participants’ opinions and beliefs about skin checking appeared to change after using the ASICA app:

I would just say you’re, you’re far more aware of it, checking your skin and stuff now as what you ever, maybe more so now, than what I was previous.

Male, 54 years, intervention

Overall, for members of the intervention group, using the ASICA app appeared to increase positive attitudes toward and frequency of practice of TSSE.

#### Feelings About Skin Checking in Both Intervention and Control Groups

Although it was possible that frequent skin checking could function to increase or heighten worries about melanoma recurrence, there was little evidence within the transcripts that feelings and emotional responses to skin checking were especially powerful. For most, it appeared to be a straightforward and nonemotive practice.

Of those who did, some respondents in both the intervention and control groups expressed a lack of confidence in their own ability to correctly self-check and identify changes that may be indicative of recurrence, suggesting that they saw this as a health professional’s role. This view tended to be expressed more by younger respondents:

I feel more happier if there’s someone told me that, you know, everything is right with my skin, and that’s the words coming from the specialist, and not me after checking my skin on the app, so you know.

Male, 39 years, intervention

One intervention group participant suggested that before using ASICA, their skin checking had been an ad hoc activity to reassure themselves when they became worried about something. Another intervention participant implied that ASICA worked well for them, but only because they were already confident in their ability to check their skin. None of the participants stated that they felt the need to use the tablet provided for skin checks more regularly than once a month:

[Before starting using the ASICA app] I just kind of tended to keep an eye on things generally, you know, without kind of saying, oh I must do it this week or whatever, I just do it whenever, whenever it came to mind, but quite often I would say, because I was kind of worried about it.

Female, 47 years, intervention

### Theme 2: Family and Friends’ Input Into Skin Checking—Opinions From Both Intervention and Control Groups

Previously, one of the perceived difficulties with skin checking was that it was difficult to perform thoroughly alone. Not all participants received regular help with skin checking, although approximately three-quarters did:

The bit I can’t see is my back, so my husband checks that for me regularly.

Female, 53 years, intervention

Views on the absolute necessity of having assistance varied. Two participants perceived themselves as being unable to complete effective TSSE, particularly when checking their backs, without the support of another person. In contrast, other 2 mentioned completing it alone (eg, using a mirror), and 1 saw no barrier to other people doing the same. Clearly, individual experiences, circumstances, and capabilities were influential in determining attitudes toward this aspect of skin checking:

I find quite difficult, well I, well I sometimes don’t get that all done actually, when I’m on my own, no.

Female, 73 years, intervention

The most difficult is the scalp, because you obviously can’t see that, so but I’ve got short hair, so it’s not too bad, the rest you can pretty much do yourself, with a mirror.

Female, 62 years, intervention

### Theme 3: Input of Health Professionals Outside the ASICA Trial—Experiences From Both Intervention and Control Groups

The involvement of health care professionals outside the trial varied according to the demographic and health status of the interviewees. Some had been attending scheduled melanoma follow-up appointments in the hospital during the study period, others had completed follow-up appointments and had seen only their general practitioner as required, whereas others had received no health professional contact at all:

They’re remarkably busy as, as you would expect and so I was discharged from dermatology and advised that if I had any concerns that I would, should see my GP.

Female, 49 years, control

Participants generally perceived difficulties and barriers to obtaining appointments for their skin in both primary and secondary care:

She had to refer me to a GP which, and she, and I had to wait three weeks to see the GP.

Female, 73 years, intervention

Two interviewees discussed having a lack of confidence in their GP’s expertise on skin and the corresponding ability to reassure them when concerns arose. However, overall, approximately half reported that they were highly satisfied with the care they were receiving. Approximately a quarter of the participants expressed a preference for receiving specialist care rather than care from a GP:

Prefer to speak with the, some kind of professionals, someone like a dermatologist, than just checking myself, because you know, if you see your, your body on a daily basis, it’s difficult notice that any changes or something is going on, especially on the back of my body.

Female, 48 years, control

### Theme 4: Ideas Around Technology From Both Intervention and Control Groups

Participants in the sample were relatively experienced in using technology, with more than three-quarters of the participants saying that they used digital devices and apps every day. Most older participants were already familiar with technology and did not perceive it as a barrier to participation because they reported receiving good training at the start of the study. Two participants who were not as keen on using technology in health care were from the control group, both female, rurally based, and aged >70 years. Both appeared relaxed about their skin and viewed ongoing structured skin checking and follow-up as potentially burdensome and stressful:

I don’t know, I don’t know if it’s an app or not. Well I mean, I’ve got two apps on my phone today. One of which works and one doesn’t yet.

Female, 76 years, control

Oh gosh, daily, many, many times a day.

Female, 48 years, control

The use of other skin-checking apps was rare, and most participants were unaware of their existence. One participant in the control group had used another skin-checking app but found it ineffective; however, using ASICA intrigued them. Previous experience of using digital technology specifically for health care was limited within the sample, but there was a general agreement that technology offered promise for more efficient care in the future. One described ASICA as “like a real innovation” (Male, 39 years, intervention).

However, there were contrasting views on the direction of the influence of technology on health care in the future. Generally, younger participants and those from rural settings expressed the most enthusiasm. One participant perceived efficiencies of care, whereas another clearly felt that technology could not substitute for personal face-to-face care:

We use mobile technology and phones and apps so much these days, I think it’s the way to go. It prevents you sort of wasting the GP’s time.

Female, 62 years, intervention

I don’t rely...on the ASICA thing to actually keep me ... up to date, if I’ve got a, really concern, I would contact the hospital. Think it’s nice as a backup, but when it comes to actually examining moles that could be cancerous I think that still needs to be done by [specialist], when they check me, they’re using a specialist eye glass.

Female, 53 years, intervention

Confidentiality and data protection are clearly important considerations when using technology to deliver health care although, notably, only 1 of the interviewees directly volunteered concerns about confidentiality. The concern was about their own device security rather than data misuse:

I wouldn’t want any images being stored and easily accessible on my device, so happy if it’s all kind of encrypted and locked away by passwords, so that would be a big deal for me in terms of where any data was stored...no problem with it being uploaded to external storage, but I would have an issue with it being stored locally.

Female, 39 years, control

When asked about the qualities that effective digital health care apps should possess, participants reported that they should be coherent and consistent in purpose, straightforward, uncomplicated, and easy to use. It was also said that it is important that apps contain enough information for them to be used effectively, and that they signpost the user to further help if needed.

### Theme 5: “Nuts and Bolts”—the Practical Experience of the ASICA Trial—Opinions of the Intervention Group Only

Participants in the intervention group who had used the ASICA app reported few technical problems. Most importantly, there did not appear to be any particular issues experienced and associated with a single demographic characteristic. One significant issue was that 1 participant stated that they had not received monthly prompts through SMS text messages (ID: *11018, male, 39 years, int*). Furthermore, 2 participants reported that some photographs appeared missing from their digital skin maps. The hardware provided (Samsung Galaxy tablets) did appear to have created technical problems with slow functioning and charging difficulties. The operating system of the tablet was identified as a barrier to its use:

The Samsung app’s a bit slow and cumbersome and just not quite up to speed really is what the ipad is.

Female, 62 years, intervention

Another issue expressed was the tablet’s camera, a key requirement of ASICA for reporting. Just under a quarter of the intervention group interviewed submitted poor quality photos [26], hindering interpretation and assessment of the photographed lesion by the Dermatology Nurse Practitioner. This then required the submission of further photos, usually from their smartphones, to enable the assessment to be completed:

They didn’t get a good enough picture using the tablet, and so when, the guy phoned me back the next day, he said can you use your smart phone to take a picture, and then send through, because just because the, what he could see, like I’m not really sure what I’m looking at.

Female, 48 years, intervention

In general, most participants viewed participation in the ASICA trial positively. Many expressed interest in participating as having an altruistic intent to improve care for others rather than to expect a personal benefit.

### Theme 6: ASICA—Impact, design, and usability—What Did the Intervention Group Think?

#### Feelings About Using ASICA

In the ASICA trial, the primary outcomes measured in both groups were psychological well-being and quality of life measured using the melanoma worry scale, Hospital Anxiety and Depression Scale, and EQ-5D [29]. A mixture of views was expressed on these points by the ASICA users interviewed here and are found in subsequent sections.

Two participants indicated an initial increase in concern when they began using ASICA but suggested that this decreased as they continued to use it. Another participant stated that the app did not change their worry level but suggested that this may not have been the case if they did not have regular access to specialists as part of the ongoing scheduled melanoma follow-up. In contrast, some participants reported finding that they became less anxious when they started using ASICA and were positive about the app, enabling people who are likely to worry about the opportunity for greater reassurance compared with less structured alternatives. Importantly, it did not seem that the arrival of email or text prompts reminding participants to perform a TSSE led to an increase in anxiety:

I think having the app kind of brought it back for a while, and I think looking at the pictures didn’t really help that, to start with, but I kind of came okay again, with kind of regularly using it.

Female, 47 years, intervention

I don’t worry too much, because I am still under the hospital.

Female, 53 years, intervention

#### Barriers to and Facilitators of Engaging With ASICA

It appeared that those who had completed follow-up and were no longer receiving regular hospital appointments, especially liked using ASICA. One reported that it gave them a sense of “no longer being off the radar.” Conversely, 1 intervention group participant said they relied on and engaged less with ASICA after being initially enthusiastic, because they still had ready access to a dermatologist and felt they were receiving enough input from them. ASICA users also reported that it was more convenient to use the app to submit concerns about their skin, rather than having to travel and park at the hospital to attend outpatient appointments. There was a sense from some, however, of a certain unwillingness to completely trust the ability of the app to detect something as serious as cancer. Another respondent underlined that the validity of ASICA had not been proven because it was novel and untested. For this individual, the concern was mitigated by the safety net of still being in formal follow-up. The interviews were completed before the COVID-19 pandemic; therefore, it was not possible to determine whether the experience of the pandemic had an influence on participants’ views.

#### Areas for ASICA Improvement

The layout, design, and functionality of the app were generally well-received, and only a few participants offered feedback for improvement. The addition of a skip button to prevent skin-checking instructional videos from playing in full each time was mentioned by 2 participants as a time-saving measure. Furthermore, the ability to pinch and zoom on skin map digital photos and clearer orientation is also recommended. The largest area of feedback was that the app should be made available for different operating systems and devices, specifically Apple, so that future users could use the app on their own devices rather than the 17.8-cm tablets, which were reported to be awkward to use by some:

You have to watch all the videos before you can just go into actually doing the, the, putting in your data, and that, so and that drives me mad.

Female, 48 years, intervention

I hate that ruddy bit of kit I’m supposed to sort of keep in communication with you, it’s a Samsung thing, I’m an Apple man, so I hate the damn thing, so that gets me frustrated, because I don’t understand.

Male, 86 years, intervention

## Discussion

### Summary of Main Findings and Comparison With the Literature

Melanoma follow-up is extensively practiced and, in recent years, has become more important because improved treatments mean that detecting new primaries and recurrences at an early stage offers the best outcomes for people with melanoma [36]. There is growing evidence on the value of TSSE as part of follow-up and how it is perceived and best performed by patients [37].

This nested qualitative evaluation showed a range of experiences, behaviors, beliefs, and feelings among people previously treated for melanoma with respect to their skin, skin checking, and technology in improving TSSE awareness, practice, and quality. Most participants stated that they checked their skin regularly but that using the ASICA intervention increased the frequency and consistency of checking and supported a more systematic approach to their skin-checking practices. Most participants did not believe that ASICA had changed their feelings around skin checking, but it had raised their awareness and changed their own skin-checking behaviors for the better. Some ASICA users reported that the intervention improved their confidence regarding when and how they checked their skin.

Few participants managed skin checking alone, and opinions on the role of health professionals in satisfactory skin checking were mixed. Interestingly, follow-up and melanoma care by GPs were viewed positively in a study in the United Kingdom, in contrast to some negative views expressed here [38]. GP follow-up has been previously viewed as a low-tech solution to the challenge of increasing melanoma follow-up burden. Now, perhaps, ASICA, with its facility of rapid access to a remote dermatology specialist, offers a more elegant solution consistent with the priorities of melanoma survivors. Participants in this study valued specialist input and the opportunity to contact a specialist during scheduled follow-ups.

A systematic review of 15 studies further identified reassurance from qualified professionals as reducing worry [39]. This was also found in the present data, with participants feeling reassured that a specialist nurse practitioner reviewed their submitted concerns and images. The added dimension in this study was that anxiety may peak when commencing TSSE (declining thereafter as skin checking becomes more routine), a point that could be borne in mind when developing training and preparing people to undertake TSSE. In contrast, several participants viewed the app as a means of providing rapid reassurance when concerns arose.

Several studies have demonstrated that there is anxiety after melanoma around attendance at follow-up appointments [40]. Our qualitative data consolidate the findings of an earlier qualitative study that identified structured skin checking as a reassurance technique for those who experienced anxiety following a melanoma diagnosis [41]. These investigators also suggested that low self-confidence about skin checking was a barrier to achieving this benefit; therefore, our respondents’ view that ASICA improved their confidence in performing TSSE is very encouraging [41]. Overall, the use of ASICA did not appear to result in adverse psychological effects. Some participants suggested that ASICA temporarily and briefly increased their anxiety about skin checking at the start, but that this has settled over time with regular use.

This study was conducted before the onset of the COVID-19 pandemic and its disruptive effects on all aspects of cancer care. However, even then, participants were almost universally well-disposed to technology becoming an integral part of their health care, with the caveat that personal interaction can be an element of high-quality care. Most saw the potential of digital technology and the likelihood that it will be increasingly used to facilitate health care in the future.

Participants had mainly positive experiences of using the ASICA intervention and made several useful suggestions to improve usability and functionality. With the user experience in mind and aiming for an app that meets the needs of melanoma survivors that are using it, these suggestions will be incorporated into the newest version of the ASICA prototype. The hardware provided had perceived limitations, with several participants reporting issues with the camera (providing images of poor clarity) and the operating system (slow functioning and charging difficulties). There was no capacity to make adjustments during the trial. All feedback has been noted, and an updated prototype is currently being developed. The unwieldy 17.8-cm tablet was perceived by some as a major barrier to using ASICA effectively and may have contributed to lower adherence for some [27]. Adherence to the intervention was explored in a further paper [27] and it was found that baseline depression, anxiety, and low confidence (also highlighted here) predicted adherence. The means of improving adherence will be a major focus of future work.

Travel and distance have previously been recognized as barriers to participating in and benefiting from melanoma follow-up, whereas having a life partner has been cited as an important source of support for successfully completing a TSSE [39,42]. In this context, the positive views expressed by our rural participants are encouraging and support the prevailing view that remote health care represents an effective way to meet the challenges of geography in the future.

Finally, age did not appear to be a barrier to the adoption of ASICA, with older users generally reporting that it was easy to use and that the training prepared them well. This suggests that older people are sufficiently familiar with technology, and that the design of ASICA avoids an age-adoption barrier [24].

### Strengths and Limitations

Nesting qualitative interviews within the main ASICA trial enabled an in-depth exploration of participants’ beliefs about their experiences with skin checking, use of technology, and the ASICA app. It also provided access to participants who were actively thinking about and engaged with TSSE to provide qualitative data that would facilitate understanding of the quantitative results of the ASICA trial. The ASICA app was initially field-tested with 19 participants and then revised for the current trial according to the participants’ feedback [24]. The results provide invaluable insights into how digital health care is perceived and experienced by these users, which is very important given the likely increasing role of technology in delivering dermatology care in the future National Health System.

Of the 240 ASICA participants, 221 (92%) consented to be contacted for the interview. There were no striking demographic differences between the participants who consented to be interviewed and those who declined to participate. However, we recognize that this substudy has the potential for selective recruitment bias. This nested qualitative evaluation sample size of 22 participants represented almost 10% of the total number of participants in the ASICA trial. This sample was purposefully selected to include a range of demographics from 2 UK sites, with locations further assessed to assure representation of both urban and rural participants. This sampling method ensured an enhanced representativeness. Including participants from both the intervention and control groups enabled a further dimension of understanding in the analysis, enabling the attitudes and experiences of those who had experienced the intervention to be compared.

In contrast, the sample was, on average, relatively well-educated and affluent, with an underrepresentation of participants from deprived backgrounds. This is important because individuals from deprived backgrounds are likely to face more challenges in adopting digital health care [43,44] and should be given higher priority for digital health care research. There is also the possibility of volunteer bias, because those agreeing to be interviewed were likely to be interested in technology. This may be reflected in the generally positive tone of the data, along with detailed infrastructural frustrations.

Importantly, there are likely to be differences between participants who did and did not fully engage with the ASICA intervention over the course of the trial. Such differences are both theoretically and clinically relevant, indicating, respectively, who is least likely to engage with the intervention provided and who is least likely to benefit as a result. It was not possible within this qualitative study to capture the experiences of those who did not fully engage with the intervention, as these participants were the least likely to provide consent to participate in an additional interview. This issue could be explored in the future by analyzing TSSE adherence data collected during the ASICA trial to identify the characteristics of those who did not engage.

The interviews were conducted on 2 tranches by 2 different interviewers. The transcripts from both sets of interviews were then analyzed by a third researcher with support from a senior qualitative researcher and senior clinical researcher. We believe there are limitations and strengths of this approach, which is similar to the accepted practice of secondary analysis of qualitative data [45]. This enabled the data to be considered from different disciplinary perspectives and also provided an objective and consistent interpretation of the written factual data. However, this approach clearly precludes any interpretation based on a recollection of the emotional tone during particular interviews. This limitation was somewhat mitigated by FR spending considerable time immersed in the data over 3 detailed readings. Furthermore, the ongoing involvement of all members of the research team ensured agreement with the emerging themes and overall interpretation. The layered approach to the study also reduced individual researcher bias. In the context of the responses from ASICA users, this has given quite a clear sense, with respect to ASICA, of what worked well, what did not, and what needed to change.

This nested qualitative evaluation study embedded within an RCT provided findings that complement the main trial findings. With the caveat of some practical issues (mainly relating to a suboptimal digital interface as currently configured), ASICA was positively received by users who found it helpful and that it improved attitudes toward the frequency and thoroughness of a TSSE. Some participants reported an initial upswing in anxiety related to using the ASICA, but this subsequently settled over time. The system was effective and viewed positively in general, with some constructive suggestions made for improvement. The study provides important qualitative information that a digital health care intervention is an effective means of prompting, recording, and responding to structured TSSE by melanoma survivors. Furthermore, it appears that ASICA has the potential to improve future aftercare for melanoma survivors. However, the limitations of the proposed technology must be recognized. It currently works better than others. However, we have a good basis for further work to develop our prototype to enable a wider demographic range of melanoma survivors to benefit maximally.

## Appendix

Multimedia Appendix 1 Achieving Self-Directed Integrated Cancer Aftercare qualitative study topic schedule.

Multimedia Appendix 2 Framework.

Multimedia Appendix 3 CONSORT-eHEALTH checklist (V 1.6.1).

## Abbreviations

ASICA: Achieving Self-Directed Integrated Cancer Aftercare
DNP: dermatology nurse practitioner
RCT: randomized controlled trial
TSSE: total skin self-examination

## Data Availability

The data sets generated and analyzed during this study are not publicly available because of concerns about the potential for individual participants to be identified from the data and the scope of the ethics approvals received. However, data may be available from the corresponding author upon reasonable request and subject to appropriate safeguards.
