# Supplementary material for: User Experiences in a Digital Intervention to Support Total Skin Self-examination by Melanoma Survivors: Nested Qualitative Evaluation Embedded in a Randomized Controlled Trial
Source: JMIR Dermatol. 2023 Feb 13;6:e39544. doi: 10.2196/39544 (PMC10335150; doi:10.2196/39544)
Supplement: Multimedia Appendix 1 [file derma_v6i1e39544_app1.docx]

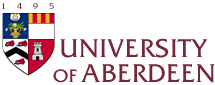


**THE ASICA RCT**

**Achieving Self-directed Integrated Cancer Aftercare (ASICA) for melanoma survivors: a nested qualitative evaluation of user experiences in a randomized trial of a digital intervention to support total-skin-self-examination by melanoma survivors.**

**Felicity Reilly1, Nuha Wani2, Susan Hall1, Heather Morgan1, Julia Allan4, Lynda Constable3, Peter Murchie1**

**TOPIC GUIDE FOR PATIENT POST RCT INTERVIEW**

The interview will be directed to some extent by the patient, but it is anticipated that the following topics will be covered.

**PREAMBLE TO BOTH GROUPS**

Thinking about the last twelve months

What are your thoughts about taking part in this study?

Prompt – what was good about it?

Prompt - What was not so good?

Have you been regularly examining your skin?

Have you had any issues with your skin?

Prompt – describe these if yes?

**FOR THE INTERVENTION GROUP**

Tell me how have you got on using the ASICA app on your tablet?

*Ease of use:*

How easy did you find it to use?

Were you able to follow the instructions on the tablet?

Was it useful to get a monthly reminder to check your skin?

Do you think the app is catering to all users?

*Information:*

Was the information accurate?

Was the information easy to understand?

Was the information concisely written?

Was the language written at an appropriate level (i.e. no jargon)?

Were there any spelling/grammatical errors?

Are there sources for the information given (e.g. link to original stats)?

*Design:*

Is the design of the app cohesive?

Was the app intuitive to use?

*Support:*

Was there adequate access to technical support?

Did you use the handbook?

*Security:*

Have you any concerns about confidentiality while using the app?

Is the privacy policy easily accessible in the app?

*Problems:*

Did you have any problems/technical difficulties with it?

Did anything prevent you from using/getting the most out of the app?

Were there any aspects of the app design that were confusing?

Tell me about your use of apps in general?

Do you use apps other than ASICA on your phone/tablet? If so, which of the following:

Social media?

Gaming?

Health and fitness?

Productivity?

Tell me about your use of melanoma self-monitoring apps?

Have you used other melanoma self-monitoring apps?

If yes, how does ASICA compare?

Tell me about how you self-monitored your skin before using ASICA?

Did you check your skin for any changes (regularly or just occasionally)?

What method did you use to self-monitor your skin before ASICA (e.g. tutorials online, instructions from dermatologist/GP)?

Before ASICA, did you use your phone/tablet to aid you in self-monitoring your skin e.g. set reminders?

Did you think that using an app to aid in skin self-monitoring would have been beneficial to you before the study? (Have you changed your mind now?)

Were there any occasions when you were concerned about your skin?

Tell us about what happened?

Did you use ASICA to communicate concerns to the nurse?

If yes - what was the result?

Thinking about your experience of using ASICA?

What were the best aspects of using ASICA?

What were the worst aspects of using ASICA?

Do you have any suggestions for how we could make ASICA better?

Does the app fulfil its purpose?

Do you now find it easier to self-monitor your skin with the aid of ASICA or was it easier before?

Does using the app give you more peace of mind about monitoring your skin?

Would you recommend the app?

**FOR THE STANDARD CARE GROUP**

Do you check your skin for any changes?

Prompt - Regularly or just occasionally?

Tell me about your methods of self-monitoring your skin?

What method do you use to self-monitor your skin (e.g. tutorials online, instructions from dermatologist/GP)?

Does anyone help you check your skin?

Do you use your phone/tablet to aid you in self-monitoring your skin e.g. set reminders?

Would getting a monthly reminder text or email help you to remember to check your skin?

If you noticed any changes would you see your GP or wait until your next hospital appointment?

Tell me about your use of apps in general?

Do you use apps on your phone/tablet? If so, which of the following:

Social media?

Gaming?

Health and fitness?

Productivity?

Tell me about your use of melanoma self-monitoring apps?

Have you used melanoma self-monitoring apps?

Do you think that using an app to aid in skin self-monitoring would be beneficial to you?

Is there anything that we haven’t covered you would like to say about being involved in the study?

**EVALUATION TOOL FOR HEALTHCARE SMARTPHONE APPLICATIONS**

| **Contents** | | | | | |
| --- | --- | --- | --- | --- | --- |
| **Accuracy** | Information provided in the healthcare app is accurate (there is no inaccurate information). | 0 | 1 | 2 | 3 |
|  | Clear information is provided in the healthcare app. | 0 | 1 | 2 | 3 |
| **Understandability** | The healthcare information in the app is readily understandable. | 0 | 1 | 2 | 3 |
|  | The healthcare information in the app is explained in everyday terms. | 0 | 1 | 2 | 3 |
|  | People in general can easily read the healthcare information provided in the app. | 0 | 1 | 2 | 3 |
| **Objectivity** | Professional healthcare information is provided. | 0 | 1 | 2 | 3 |
|  | Healthcare information is provided systematically. | 0 | 1 | 2 | 3 |
|  | There is an indication that the healthcare information is cited from authoritative sources. | 0 | 1 | 2 | 3 |
|  | Medical experts provide the healthcare information. | 0 | 1 | 2 | 3 |
| **Interface Design** | | | | | |
| **Consistency** | The app has coherence in terms of color, configuration, and expression method. | 0 | 1 | 2 | 3 |
|  | Icon arrangement is in harmony with the whole app design. | 0 | 1 | 2 | 3 |
|  | Icons are categorized coherently in the app. | 0 | 1 | 2 | 3 |
| **Suitability of Design** | Arrangement of contents is well organized enough to be sequentially accessible and logically understandable. | 0 | 1 | 2 | 3 |
|  | The meaning of each icon is clearly expressed. | 0 | 1 | 2 | 3 |
|  | The app has highly readable typography. | 0 | 1 | 2 | 3 |
|  | Visual elements do not confuse users. | 0 | 1 | 2 | 3 |
|  | The structure of the app can be clearly grasped. | 0 | 1 | 2 | 3 |
| **Accuracy of Wording** | Instructions are told in a concise manner. | 0 | 1 | 2 | 3 |
|  | Instructions are told in a precise manner. | 0 | 1 | 2 | 3 |
|  | All words are not merely spelled correctly but also grammatically correct | 0 | 1 | 2 | 3 |
| **Technology** | | | | | |
| **Security** | The app offers information about privacy protection. | 0 | 1 | 2 | 3 |
|  | The app offers information about security policies related to personal health information | 0 | 1 | 2 | 3 |
|  | The app explained the security system for creating a safe environment for better mobile app usage. | 0 | 1 | 2 | 3 |

0 = not at all | 1 = a little | 2 = a fair amount | 3 = a lot

0-23 = poor | 24-46 = average | 47-9 = satisfactory

**DEMOGRAPHIC CHARACTERISTICS**

What is your age?

Sex

- Male
- Female
- Other

Ethnicity

- White – English/Welsh/Scottish/Northern Irish/British/Irish/Gypsy/Irish Traveller/Other
- Mixed/Multiple ethnic groups – White and Black Caribbean/White and Black African/White and Asian/Other
- Asian/Asian British – Indian/Pakistani/Bangladeshi/Chinese/Other
- Black/African/Caribbean/Black British – African/Caribbean/Other
- Other – Arab/Other

Educational attainment

- Early childhood education
- Primary education
- Lower secondary education
- Upper secondary education
- Post-secondary non-tertiary education
- Short-cycle tertiary education
- Bachelor’s or equivalent level
- Master’s or equivalent level
- Doctoral or equivalent level

Employment status

Marital status

Language

- English as first language?
