# Supplementary material for: User Experiences in a Digital Intervention to Support Total Skin Self-examination by Melanoma Survivors: Nested Qualitative Evaluation Embedded in a Randomized Controlled Trial
Source: JMIR Dermatol. 2023 Feb 13;6:e39544. doi: 10.2196/39544 (PMC10335150; doi:10.2196/39544)
Supplement: Multimedia Appendix 2 [file derma_v6i1e39544_app2.docx]

**Achieving Self-directed Integrated Cancer Aftercare (ASICA) for melanoma survivors: a nested qualitative evaluation of user experiences in a randomized trial of a digital intervention to support total-skin-self-examination by melanoma survivors.**

**Felicity Reilly1, Nuha Wani2, Susan Hall1, Heather Morgan1, Julia Allan4, Lynda Constable3, Peter Murchie1**

1. Skin Checking
   1. How people check their skin
      1. Including app use
   2. When people check their skin
      1. Prompts and triggers
      2. Location.
   3. Experience of skin checking
   4. Opinions on skin checking
   5. Feelings on skin checking
   6. Relationship between skin and skin checking and health.
2. Family and friends
   1. Other people (non-professionals) input to skin checking
   2. Relationship with people assisting with skin

1. Other health professionals outside of ASICA trial
   1. Use of health services
   2. Opinions on health service especially services for skin
2. Ideas around technology
   1. Use of apps in general
   2. Previous use of health apps
      1. Especially other skin checking apps
   3. Opinions on the use of apps in health care
   4. Impact of app on use of health services
   5. Confidentiality
   6. Opinions around what apps (especially health care) should be and do
3. ‘Nuts and Bolts’- Practical experience of ASICA
   1. Practical use of app and tablet
   2. Experience of the tablet provided
   3. Experience of the technical side of the app
   4. Experience of the other practical aspects of the trial
   5. Attitude towards science/trials
4. ASICA: the app impact, design and usability
   1. Feelings around the app
      1. Anxiety level
      2. Change in opinions on skin health due to the app.
   2. Positive aspects of the app itself
   3. Negative issues around the design of app
   4. Ideas for improvement of the app and its interface
